# *TBL2* methylation is associated with hyper-low-density lipoprotein cholesterolemia: a case-control study

**DOI:** 10.1186/s12944-020-01359-8

**Published:** 2020-08-18

**Authors:** Yang Li, Shuai Liu, Yong-Tao Wang, Han Min, Dilare Adi, Xiao-Mei Li, Yi-Ning Yang, Zhen yan Fu, Yi-Tong Ma

**Affiliations:** 1 Department of Cardiology, First Affiliated Hospital of Xinjiang Medical University, 137 Liyushan South Road, Urumqi, 830054 China; 2 Xinjiang Key Laboratory of Cardiovascular Disease Research, Urumqi, China

**Keywords:** Hyperlipidemia, Coronary artery disease, Hyper-low-density lipoproteinemia, DNA methylation, Transducin (β)-like 2 (TBL2) gene, Haplotype, CpG island

## Abstract

**Background:**

*HMGCR, SCAP, SREBF1, SREBF2* and *TBL2* are well-known genes that are involved in the process of lipid metabolism. However, it is not known whether epigenetic changes of these genes are associated with lipid metabolism. In this study, the methylation levels of the *HMGCR, SCAP, SREBF1, SREBF2* and *TBL2* genes were analyzed between samples from a hyper-low-density lipoprotein cholesterolemia (hyper-LDL) group and a control group to examine the association between the methylation levels of these genes and the risk of hyper-LDL.

**Methods:**

In this study, a case-control approach was used to explore the association between DNA methylation and hyper-LDL. The DNA methylation levels of *HMGCR, SCAP, SREBF1, SREBF2* and *TBL2* genes and 231 CpG sites in the promoter regions of these genes were measured in 98 hyper-LDL participants and 89 participants without hypo-LDL.

**Results:**

Compared with participants without hyper-LDL, patients with hyper-LDL *TBL2* gene had lower methylation levels (11.93 vs. 12.02, *P* = 0.004). The methylation haplotypes with significant abundance in the TBL2 gene are tcttttttttt (*P* = 0.034), ctttttttcct (*P* = 0.025), ctctttctttt (*P* = 0.040), ccttttttttt (*P* = 0.028), and tctttttttttttttt.

**Conclusion:**

The study demonstrates that participants with hyper-LDL have lower methylation of *TBL2*. The results suggest that DNA methylation of *TBL2* can decrease the risk for hyper-LDL in humans.

## Background

With the development of economy and changes in lifestyle, especially aging population and acceleration of urbanization, the prevalence of Coronary artery disease (CAD) is clearly on the rise which is the main cause of death [1, 2]. Several epidemiological studies have confirmed that hyperlipidemia has a significant association with CAD in the past decades [3]. Clinical studies have revealed that the prevalence of CAD increases as the low-density lipoprotein (LDL) level increases in plasma. In addition, total cholesterol (TC) ranging from 5.2 to 6.2 mmol/L lead to a threefold increase in the morbidity of cardiovascular disease [4]. Many gene mutations have been shown to be responsible for hyperlipidemia, and the main causes of hyperlipidemia are LDL receptor (LDLR) gene defects [5]. In addition, the internalization of the LDLR caused by the proprotein convertase subtilisin kexin type 9 (PCSK9) and ApoB can result in a similar phenotype [6]. Along with genetic mutations, epigenetic changes are also associated with hyperlipidemia. A large number of studies have shown that DNA methylation, as an important epigenetic mode, can regulate gene expression by changing chromatin structure, DNA conformation, DNA stability and interaction between DNA and proteins [7, 8].

Previous studies have revealed that the conversion of HMG-CoA to mevalonate is catalyzed by HMG-CoA reductase (HMGCR), which limits the process of cholesterol biosynthesis. Previous studies also have found that sterol regulatory element-binding transcription factors (SREBFs) control cholesterol homeostasis by regulating the transcription of cholesterol and lipid metabolism genes. SREBF cleavage activator protein (SCAP) and SREBFs can form a complex when the cell sterols are depleted. The complex is transported to the Golgi apparatus and triggers the amino-terminal transcriptional activation domain of SREBFs after being processed by two specific proteases. The activated complex then enters the nucleus, where it combines with the promoter region of the target gene [9, 10]. In a previous study, the transducin (β)-like 2 (*TBL2*) gene was identified as a new genetic locus affecting lipid concentration [11], which can lead to hypertriglyceridemia disease. However, how TBL2 affects cholesterol has not yet been reported.

The *HMGCR, SCAP, SREBF1, SREBF2* and *TBL2* genes are well-known genes involved in the lipid metabolism. However, it is not known whether there are any epigenetic associations in the lipid metabolism. Among the hyperlipidemias, high-density lipoproteinemia has the greatest impact on the human body and is most likely to cause various diseases. Low-density lipoprotein can cause changes in other blood lipid indicators. This study sequenced the levels of DNA methylation of *HMGCR, SCAP, SREBF1, SREBF2* and *TBL2* genes between samples from a hyper-low-density lipoprotein cholesterolemia (hyper-LDL) group and a control group to explore whether the methylation levels of these genes have relevance to risk of hyper-LDL.

## Methods

### Study population

**Fig. 1:**
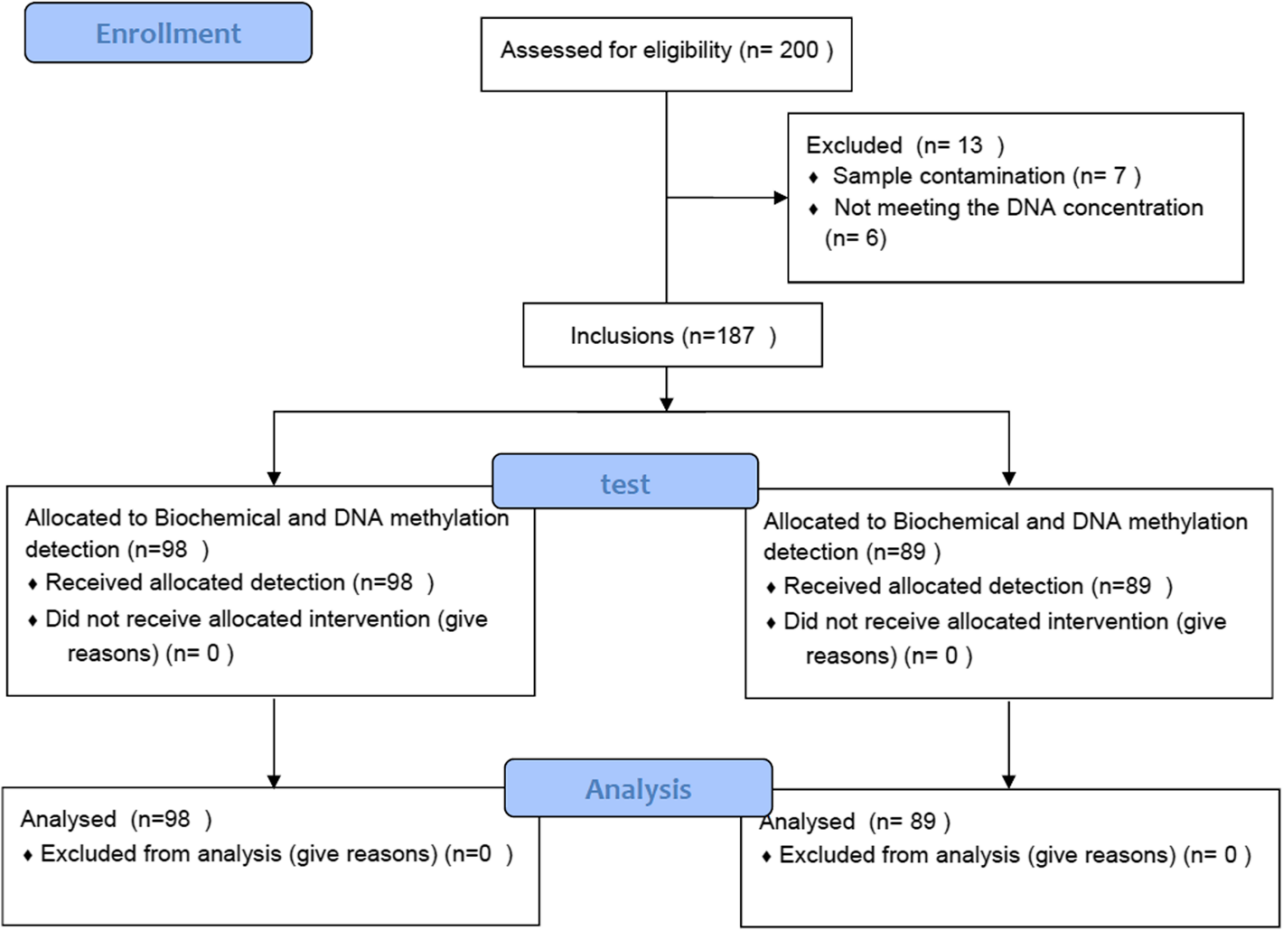
The CONSORT flow diagram of the study population. CONSORT: CONsolidated Standards of Reporting Trials

Before the research started, our team formulated a research plan on August 21, 2012, and followed it (Additional file 1). The Ethical Review Board of First Affiliated Hospital of Xinjiang Medical University approved this study (Additional file 2). The methodology of this study is based on the Declaration of Helsinki, and kept the personal information of the participants confidential. All participants voluntarily participated in this study and signed an informed consent form. The research plan was designed according to STROBE checklists-case-control (Additional file 3). Upon reviewing case-control studies on DNA methylation, the sample sizes of the case and control groups ranged from 80 to 90. Therefore, this study recruited 100 hyper-LDL participants as the hyper-LDL group and randomly enrolled 100 age- and sex-matched participants as the control group. Two hundred samples were tested for DNA methylation. This study recruited 98 participants as the hyper-LDL group and 89 participants as the control group due to sample contamination (Fig. 1). All participants were Han Chinese and enrolled from the First Affiliated Hospital of Xinjiang Medical University from 2012 to 2015.

The definition of hyper-LDL was a fasting LDL level ≥ 3.1 mmol/L. The inclusion criteria were those with completed inpatient medical records and had no history of taking lipid-lowering medicine. The exclusion criteria were as follows: 1. renal dysfunction, creatinine ≥178 μmol/L; 2. valvular disease and heart failure; 3. chronic inflammatory disease; 4. acute infectious diseases, such as severe pneumonia, cholecystitis, and acute tuberculosis; 5. autoimmune disease; 6. Tumor; and 7. acute cerebral hemorrhage or brain infarction.

### DNA isolation and epigenotyping

Fasting venous blood was collected overnight after all participants were informed instructions for attention to take biochemical detection. Blood cells from participants were collected for whole genomic DNA extraction using commercial kits (TIANGEN Biotech, Beijing, China). Whole genomic DNA was diluted with 75% ethanol to final concentration (more than 10 ng/mL) for sequencing and methylation analysis.

**Fig. 2:**
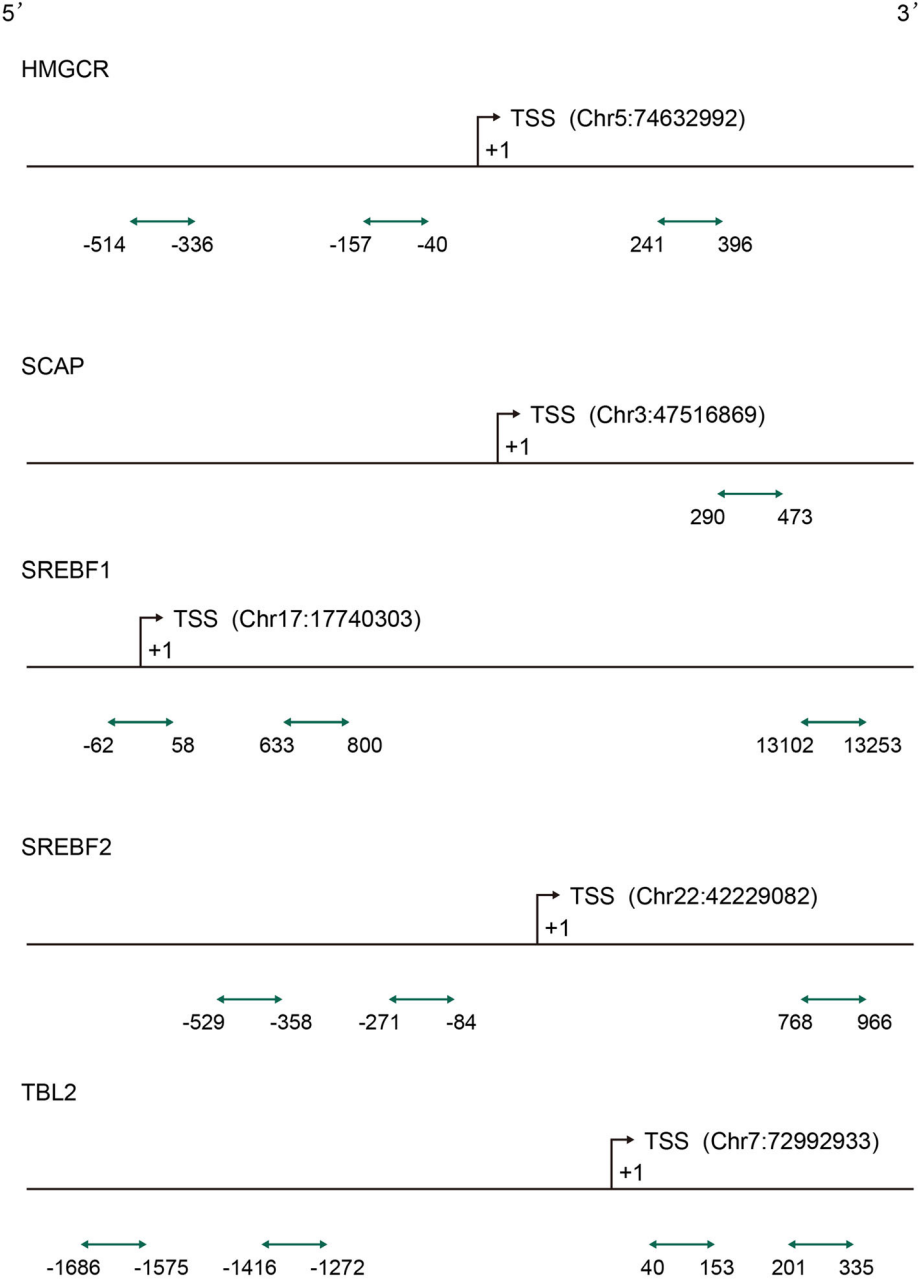
CpG sites sequenced around the promoters of *HMGCR, SCAP, SREBF1/2, TBL2.* The eligible CpG sites sequenced in this study are indicated by green lines with arrows. All regions are distributed in the CpG islands of gene promoters and first exon. *HMGCR*: HMG-CoA reductase; *SREBF*: Sterol regulatory element binding transcription factors; *SCAP*: *SREBF* cleavage activator protein; *TBL2*: transducin (β)-like 2; TSS: transcription start site

CpG islands distributed in promoters and first exon regions of *HMGCR, SCAP, SREBF1, SREBF2* and *TBL2* were captured and sequenced for methylation genotyping and analysis. The criteria of eligible CpG islands were as follows: (1) GC content not lower than 50%; (2) 200–1000 bp length; (3) The ratio of observed/expected CpG dinucleotides not lower than 0.60. Ultimately, three CpG regions of *HMGCR*, one that of *SCAP*, three that of *SREBF1/2* and four that of *TBL2* were identified in eligible CpG islands complying with the criteria and analyzed (Fig. 2). BiSulfite Amplicon Sequencing (BSAS) was applied to quantitative analysis of DNA methylation. The principle of the analysis is that sodium bisulfite gives priority to deamination of unmethylated cytosine residues to thymine, while methyl-cytosine remains unmodified. Bisulfite was used to base conversion of 1μg genomic DNA complying with EZ DNA Methylation-GOLD Kit (ZYMO RESEARCH, CA, USA). The samples were sequenced using Illumina MiSeq Benchtop Sequencer (CA, USA) after PCR amplification in target CpG islands (HotStarTaq polymerase kit, TAKARA, Tokyo, Japan) and library construction. The average coverage of all samples was >600X. The CpG regions tested were defined as the distance (in bp) between CpG sites and transcription start site (TSS). The methylated cytosine/total cytosine ratio was defined as the CpG site methylation level. The average methylation level of all detected CpG sites was defined as the gene methylation level.

### Statistical analysis

The *Shapiro–Wilk test* assessed the normality of parameters. The measurement data that met the normality assumption are represented as the means±SD. An *independent-sample T-test* was used to distinguish the hyper-LDL group from the control group. Median (interquartile range) represented the measurement data that did not suit the normality assumption and was tested with the *Mann-Whitney U test*. Further, the *Chi-square test* discriminated the categorical data, such as smoking and hypertension. Methylation levels of different genes were assessed between the hyper-LDL group and the control group by the *Mann–Whitney U test*. The *Mann–Whitney U test* was used to determine the abundance of each methylated haplotype, to screen methylated haplotypes with significant differences in abundance. The contributions of the major factors for hyper-LDL were assessed by *logistic regression analyses* with effect ratios (OR and 95% CI). IBM SPSS Statistics Version 22.0 was used to analyze data. A two-tailed value of *P* < 0.05 was considered statistically significant.

## Result

One hundred eighty-seven participants were recruited in this study. The basic characteristics and blood lipid profiles are shown in Table 1. The average age of 187 analyzed participants was 59.93 ± 10.93 years, and 61 (32.6%) were male. The study population have 101 (54%) with hypertension, 144 (77%) with CAD and 46 (24.7%) with diabetes. The hyper-LDL group had a higher prevalence of hypertension (63.3 vs. 43.8%, *P* = 0.008) and higher TC level (5.21 ± 1.03 vs. 3.57 ± 0.70 mmol/L, *P* = 0.001), TG level (1.76(0.51–5.32) vs. 1.24(0.35–6.21) mmol/L, *P* = 0.001), LDL level (3.57(2.44–5.92) vs. 1.98(1–3.42) mmol/L, *P* = 0.001) and glucose level (5.78(3.41–17.85) vs. 4.84(2.53–13.54) mmol/L, *P* = 0.001) (Table 1).

**Table 1:** Comparison of basic characteristics between participants with and without hyper-LDL

Characteristics	With Hyper-LDL (*n* = 98)	Without Hyper-LDL (*n* = 89)	*P value*
**Age, years (means ± SD)**	59.46 ± 11.12	60.45 ± 10.75	0.538
**Male, n (%)**	31 (31.6%)	30 (33.7%)	0.762
**Hypertension, n (%)**	62 (63.3%)	39 (43.8%)	0.008
**Diabetes, n (%)**	29 (29.9%)	17 (19.1%)	0.088
**CAD, n (%)**	65 (73%)	79 (80.6%)	0.219
**TC, mmol/L (means ± SD)**	5.21 ± 1.03	3.57 ± 0.70	0.001
**TG, mmol/L, median (minimum - maximum)**	1.76 (0.51–5.32)	1.24 (0.35–6.21)	0.001
**HDL, mmol/L (means ± SD)**	1.13 ± 0.25	1.12 ± 0.33	0.855
**LDL, mmol/L, median (minimum - maximum)**	3.56 (3.11–5.92)	1.98 (1–3)	0.001
**Glucose, mmol/L, median (minimum - maximum)**	5.78 (3.41–17.85)	4.84 (2.53–13.54)	0.001
**GSP, mmol/L (means ± SD)**	2.34 ± 0.46	2.27 ± 0.41	0.263
**Creatinine,** μ**mol/L (means ± SD)**	72.53 ± 22.87	71.94 ± 17.72	0.848
**Smoking, n (%)**	38 (38.8%)	25 (28.1%)	0.123

*CAD* Coronary artery disease, *TC* Total cholesterol, *TG* Triglyceride, *HDL* High-density lipoprotein, *LDL* Low-density lipoprotein, *GSP* Glycated serum protein

A total of 231 CpG sites were identified as methylation sites (44 in *HMGCR*, 16 in *SCAP*, 64 in *SREBF1*, 47 in *SREBF2* and 60 in *TBL2*) according to the measurement results of the target regions (Additional file 4 shows the detailed information of each site). In these 231 CpG islands detected, the statistical differences in methylation sites between the two groups of participants are shown in Table 2. The average methylation levels of CpG sites measured within *HMGCR, SCAP,* and *SREBF1/2* were not significantly correlated with hyper-LDL. However, the methylation level of *TBL2* was significantly related to hyper-LDL (Table 3).

**Table 2:** CpG site methylation of candidate genes between two groups

CpG region	CpG site	Methylation levels (median %, minimum - maximum)		*P value*
		With Hyper-LDL	Without Hyper-LDL	
***TBL2*–1**	37	1.45 (0.76–1.94)	1.37 (0.67–2.68)	0.027
***TBL2*–2**	33	13.42 (11.34–15.05)	13.7 (12–15.27)	0.005
	37	11.23 (9.17–13.14)	11.58 (8.54–13.49)	0.032
	89	26.80 (22.44–32.06)	27.78 (22.14–31.03)	0.0004
***TBL2*–3**	40	2.13 (1.08–3.42)	1.91 (0–6.67)	0.0004
	50	2.03 (1.18–3.14)	1.77 (0–3.18)	0.0001
	54	2.49 (1.21–4.01)	2.33 (0–10.00)	0.042
	65	1.44 (0.67–2.46)	1.35 (0–3.12)	0.015
	74	1.47 (0.87–2.71)	1.38 (0–2.85)	0.009
	78	1.64 (0.88–2.52)	1.50 (0–2.98)	0.028
	88	1.23 (0.55–2.02)	1.13 (0–14.29)	0.004
	135	0.84 (0.20–2.01)	0.72 (0–4.00)	0.023
	144	0.74 (0.16–1.76)	0.67 (0–5.00)	0.036
***HMGCR*-1**	116	0.83 (0–1.81)	0.70 (0–2.67)	0.028
***HMGCR*-2**	51	0.68 (0.19–1.68)	0.62 (0.17–2.12)	0.019
	55	0.86 (0.35–1.62)	0.95 (0.36–2.18)	0.009
***HMGCR*-3**	37	0.51 (0–1.75)	0.64 (0–1.57)	0.013
	182	1.09 (0–2.56)	0.90 (0–6.36)	0.006
***SCAP***	33	0.00 (0–6.67)	0.00 (0–10)	0.005
***SREBF1*–1**	19	3.24 (0–5.21)	2.80 (0–5.56)	0.0001
	25	0.72 (0–2.25)	0.55 (0–3.96)	0.014
	36	0.74 (0–2.06)	0.60 (0–2.12)	0.012
***SREBF1*–2**	154	0.79 (0–2.44)	1.00 (0–3.26)	0.006
***SREBF1*–3**	28	0.49 (0–2.42)	0.30 (0–3.19)	0.006
***SREBF2*–1**	101	7.74 (0–11.01)	7.53 (4.24–10.64)	0.021
***SREBF2*–2**	123	0.92 (0.08–2.6)	0.79 (0–2.86)	0.039
	157	2.60 (0.96–5.01)	2.19 (0–6.88)	0.004
***SREBF2*–3**	112	1.23 (0.12–3.17)	0.96 (0–3.18)	0.003
	213	1.13 (0.15–2.98)	0.92 (0–3.9)	0.012

*HMGCR* HMG-CoA reductase, *SREBF* Sterol regulatory element-binding transcription factors, *SCAP SREBF* cleavage activator protein, *TBL2* Transducin (β)-like 2

**Table 3:** DNA methylation level of candidate genes between two groups

Gene	AMD(%)	*P value*
***HMGCR***	0.01	**0.848**
***SCAP***	0.19	**0.147**
***SREBF1***	0.02	**0.186**
***SREBF2***	0.02	**0.51**
***TBL2***	−0.26	**0.004**

*AMD* Average level of methylation differences = the average methylation level of hyper-LDL group - the average methylation level of control group, *HMGCR* HMG-CoA reductase, *SREBF* Sterol regulatory element-binding transcription factors, *SCAP SREBF* cleavage activator protein, *TBL2* Transducin (β)-like 2

The methylation levels of candidate genes compared between two groups are shown in Table 2. Participants in the hyper-LDL group had lower methylation levels (11.93% vs. 12.02%, *P* = 0.004). The methylation haplotypes with significant abundance in the *TBL2* gene were tcttttttttt (*P* = 0.034), ctttttttcct (*P* = 0.025), ctctttctttt (*P* = 0.040), ccttttttttt (*P* = 0.028), tctttttttttttttt (*P* = 0.019), tttttttttttttttc (*P* = 0.031) and tttttttttttttctt (*P* = 0.015) (Table 4).

**Table 4:** The haplotypes of related genes between the two groups

Gene	Haplotype	*P value*
***HMGCR***	ttttttctttttt	0.004
	ttttttttttttttct	0.007
	tctttttttttttttt	0.037
***SREBF1***	cttttttttttttttttttttttt	0.009
	tttctttttttttttttttttttt	0.024
	ttttcttttttttttttttttttt	0.007
	tttttttttttttctttttttttt	0.025
	tttttttttttttctttttttttt	0.048
	tttttctttttttttt	0.034
***SREBF2***	ttttttttcttt	0.011
	ttttttttttttcttt	0.035
***TBL2***	tcttttttttt	0.034
	ctttttttcct	0.025
	ctctttctttt	0.04
	ccttttttttt	0.028
	tctttttttttttttt	0.019
	tttttttttttttttc	0.031
	tttttttttttttctt	0.035

*HMGCR* HMG-CoA reductase, *SREBF* Sterol regulatory element-binding transcription factors, *TBL2* Transducin (β)-like 2

This study performed univariate and multivariate *logistic regression analyses* with effect ratios (OR and 95% CI) to evaluate the contributions of risk factors to hyper-LDL. The DNA methylation of *TBL2* is a protective factor for hyper-LDL (Table 5). Participants with high levels of methylation of *TBL2* have a 78% lower risk of hyper-LDL (*P* = 0.017 OR = 0.221 95% CI = 0.064–0.765). In this study, TG (*P* = 0.003 OR = 1.744 95% CI = 1.211–2.511) and glucose (*P* = 0.014 OR = 1.26 95% CI = 1.049–1.513) value are treated as continuous variables in the logistic regression model to manifest they are independent risk factors for hyper-LDL. Participants with higher blood glucose or TG levels increase 26 and 74% risk to develop hyper-LDL independently.

**Table 5:** *Logistic regression analysis* for risk factors that could affect lipid metabolism

Variable	Univariate		Multivariate	
	OR	*P value*	OR	*P value*
***TBL2***	0.203 (0.06–0.686)	0.01	0.221 (0.064–0.765)	0.017
**Sex**	0.910 (0.493–1.678)	0.762		
**Age**	0.992 (0.966–1.018)	0.535		
**Smoking**	0.617 (0.333–1.141)	0.124		
**Hypertension**	0.453 (0.251–0.814)	0.008	0.651 (0.341–1.245)	0.195
**Diabetes**	0.554 (0.279–1.098)	0.09		
**Creatinine**	1.001 (0.987–1.016)	0.847		
**Glucose**	1.335 (1.129–1.579)	0.001	1.26 (1.049–1.513)	0.014
**GSP**	1.463 (0.753–2.843)	0.262		
**TG**	1.767 (1.269–2.459)	0.001	1.744 (1.211–2.511)	0.003
**HDL**	1.098 (0.404–2.988)	0.854		

*TBL2* Transducin (β)-like 2, *TC* Total cholesterol, *GSP* Glycated serum protein, *HDL* High-density lipoprotein

## Discussion

This study confirms that the increased DNA methylation in *TBL2* decreases the susceptibility of hyper-LDL. DNA methylation is a kind of pre-transcriptional modification that is characterized by specifically adding methyl groups to a nucleotide. DNA methylation regulates gene expression and maintains genomic integrity by cooperating with modified nucleosome proteins [12]. CpG islands are distributed unevenly throughout the genome where DNA methylation mainly occurs [13–15]. In vivo, approximately 80% of CpGs are methylated in normal healthy cells [16, 17]. The enzymes that catalyze the DNA methylation are named DNA methyltransferases (DNMTs): DNMT1 essentially maintains DNA methylation during cell division by catalyzing the addition of methyl groups to cytosine, generating 5-methylcytosine (5-mC) and cytosine, while DNMT3a and DNMT3b are important for de novo methylation during the methylation process [12, 18–20]. CG methylation in promoters close to transcriptional initiation sites usually inhibits gene expression [21–23]. There are two ways that promoter methylation inhibits gene transcription: first, by physically blocking the binding of transcription factors to gene promoters, and second, by binding to the methyl-CpG-binding domain, which recruits repressive machinery, such as histone and chromatin modifiers, to the loci that cause chromatin compaction [24]. Biologically, because they act as docking sites for methyl-binding proteins, methylated CpG islands are markers of gene suppression. In fact, they can produce spatial barriers that specifically bind transcription factors to gene promotors, recruit transcription inhibitors or block activation protein binding [8, 25].

In a previous study, the *TBL2* gene was identified as a genetic locus affecting lipid concentration, which can lead to hypertriglyceridemia and increase the risk of coronary artery disease [11, 26–31]. The *TBL2* gene is located in chromosome 7q11.23, and the resulting protein has five WD40 domains [32]. The WD40 repeat protein TBL2 was found to be a candidate transitional epithelial response protein 1 (TERE1)-interacting protein. The TERE1-TBL2 complex may be related to the metabolism of cholesterol [33]. Previous studies have demonstrated that abnormal expression of TERE1 or TBL2 can affect intracellular cholesterol levels [34]. It was also found that TERE1 directly interacts with HMGCR [35]. Schnyder’s Corneal Dystrophy is caused by TERE1 mutations, which have been demonstrated to have an influence on TERE1 interaction with APOE [34, 36] and with HMGCR [35]. In this study, the DNA methylation level of *TBL2* decreased in participants with hyper-LDL, which generally inhibits gene expression. The upregulation of *TBL2* gene expression, which is positively associated with the TERE1-TBL2 complex, may affect the expression of genes such as *APOE* and *HMGGR* and can ultimately lead to downregulation of cholesterol.

**Fig. 3:**
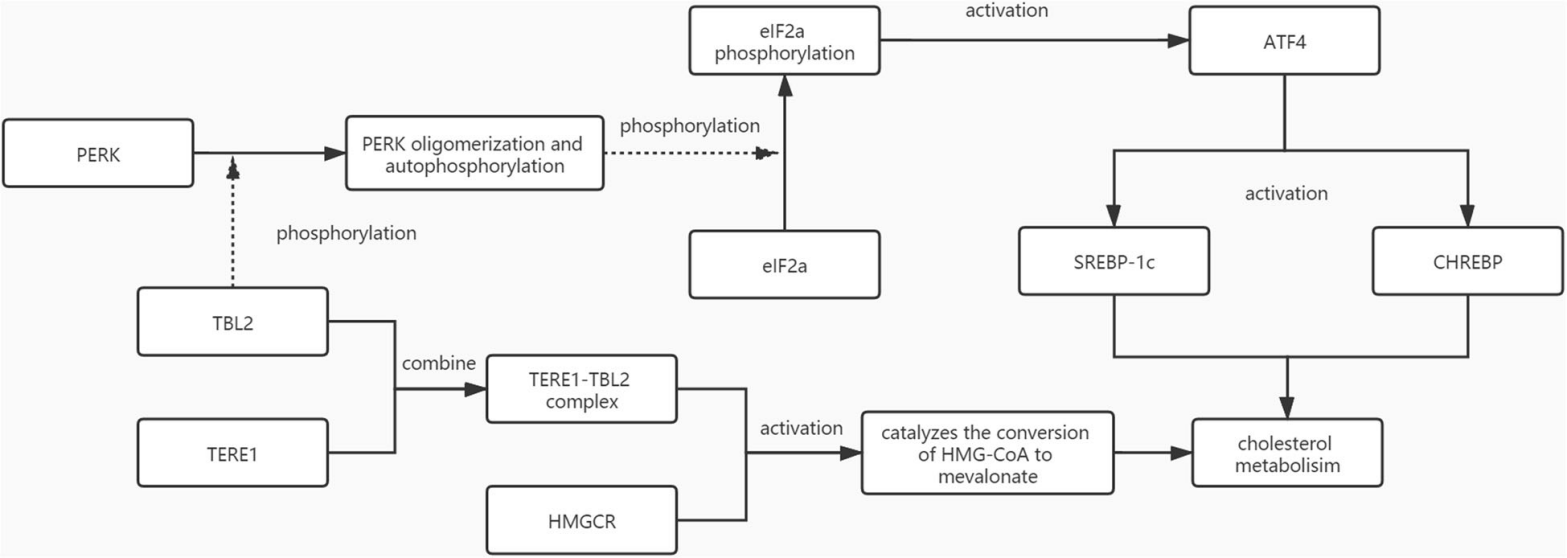
Mechanism of the role of TBL2 in hyper-LDL. *BL2*: Transducin (β)-like 2; *TERE1*: Transitional epithelial response protein 1; *HMGCR*: HMG-CoA reductase; *SREBP*: Sterol regulatory element-binding transcription factor protein; *CHREBP*: Carbohydrate response element-binding protein; *PERK*: PKR-like ER-resident kinase; *eIF2a*: Eukaryotic Initiation Factor 2 alpha; *ATF4*: Activating transcription factor 4

Previous studies have found that TBL2 can regulate the PKR-like ER-resident kinase (PERK) pathway. The TBL2 protein is localized in endoplasmic reticulum (ER) and interacts with PERK with the binding of the WD40 domain to the 60S ribosome subunit. Under ER stress, the TBL2-60S ribosome complex mediates the translation of specific proteins that can activate PERK oligomerization and autophosphorylation [37–41]. Activated PERK then phosphorylates eukaryotic initiation factor 2 alpha (eIF2a) of Ser-51 [42, 43]. Under the condition of eIF2a phosphorylation, most RNA transcription was inhibited, while the translation of RNA represented by activating transcription factor 4 (ATF4) was enhanced [44]. Previous studies also have found that TBL2 can directly affect the expression of ATF4 by binding the WD40 domain to *ATF4* mRNA [45]. It has been reported that ATF4 is related to lipid metabolism. Kun-Yun Yeh et al. found that overexpression of *ATF4* in zebrafish increased lipid accumulation in blood vessels [46]. Chunxia Wang et al. found that in *ATF4*-knockout mice, fat mobilization increased and fatty acid synthesis decreased. They identified that ATF4 can regulate lipid metabolism [47]. Previous studies have also found that the absence of ATF4 causes an increase in free cholesterol in the liver but a decrease in serum cholesterol [48]. In addition, ATF4 deficiency reduced the accumulation of triglycerides and the expression of SREBP-1c and CHREBP. It is further speculated that ATF4 affects lipid metabolism by affecting *SREBP-1c* and *CHREBP* genes [49]. In conclusion, the increased expression of TBL2 caused by the decrease of methylation level leads to the activation of the PERK pathway, which leads to the increase of cholesterol levels (Fig. 3).

### Study strengths and limitations

There are several strengths in this study. First, this study first found DNA methylation levels and haplotypes at some CPG sites of TBL2 are associated with hyper-LDL. Second, Although the mechanism of candidate genes (HMGCR, SCAP, SREBF1/2) in lipid metabolism have been studied clearly, this study first found that some CpG sites of these genes are related to hyper-LDL. DNA methylation may participate in these genes to regulate lipid metabolism. Third, this is a random case-control study. The subject of the study is people, which has certain clinical application value. This study also has limitations. First, this study is a correlation study. Further functional studies are needed to interpret mechanisms that relate TBL2 methylation to hyper-LDL. Second, this is a single-center study, and all participants in the study were Han Chinese, which may affect the generalizability of the findings.

## Conclusion

This study demonstrates that participants with hyper-LDL have lower methylation levels of *TBL2*. The results suggest that DNA methylation of *TBL2* can decrease the risk of hyper-LDL in humans. This study found a variety of DNA methylation haplotypes with statistical differences, which can tell us how the gene is mainly methylated in hyper-LDL. These different haplotypes can help us develop some specific intercalators or activators to act on CpG sites that can replace or activate the DNA methylation of this gene to treat hyper-LDL in the future.

## Supplementary information

**Additional file 1.**

**Additional file 2: Appendix 2**. Approval report of the Medical Ethics Committee of the First Affiliated Hospital of Xinjiang Medical University

**Additional file 3: Appendix 3**. STROBE Statement—Checklist of items that should be included in reports of case-control studies

**Additional file 4.**

## Data Availability

The data sets generated and analyzed during the current study are available from the corresponding author upon reasonable request (Additional file 4).
