# Supplementary material for: TBL2 methylation is associated with hyper-low-density lipoprotein cholesterolemia: a case-control study
Source: Lipids Health Dis. 2020 Aug 18;19:186. doi: 10.1186/s12944-020-01359-8 (PMC7433086; doi:10.1186/s12944-020-01359-8)
Supplement: Supplementary file 1 — Additional file 1. [file 12944_2020_1359_MOESM1_ESM.docx]

**TBL2 methylation is associated with** **hyper-low-density lipoprotein cholesterolemia: a case-control study**

**Authors and addresses**

Li Yang^1,2†^, Liu Shuai^1,2†^, Yong-Tao Wang^1,2^, Han Min^1,2^, Dilare Adi^1,2^, Xiao-Mei Li^1,2^, Yi-Ning Yang^1,2^, Zhen yan Fu^1,2*^, Yi-Tong Ma^1,2*^

* Correspondence author

† Equal contributors

1 Department of Cardiology, First Affiliated Hospital of Xinjiang Medical University, Urumqi, China.

2 Xinjiang Key Laboratory of Cardiovascular Disease Research, Urumqi, China.

## Study aim(s)

## Exploring whether the DNA methylation of HMGCR, SCAP, SREBF1, SREBF2 and TBL2 genes are related to hyper-low-density lipoprotein cholesterolemia(hyper-LDL).

## Study population

We recruited 98participants as hyper-LDL group and 89 patients as control group. All participants were Han Chinese who recruited from the First Affiliated Hospital of Xinjiang Medical University from 2012 to 2015.The definition of hyper-LDL was the level of LDL ≥3.1

The inclusion criteria were those with completed Inpatient medical records and without taking lipid-lowering drugs.

Exclusion criteria : 1. renal dysfunction 2. valvular disease and heart failure 3. chronic inflammatory disease 4. Acute infectious diseases such as severe pneumonia, cholecystitis, acute tuberculosis, etc. 5. Autoimmune disease 6. tumor 7. Acute cerebral hemorrhage or brain infarction.

## DNA isolation and epi-genotyping

Venous blood samples were taken from participants in the morning after fasting overnight for biochemical detection and methylation analysis.

We selected CpG islands located in the proximal promoter of HMGCR, SCAP, SREBF1,SREBF2 and TBL2 for methylation analysis.

The criteria as follows: (1) 200 bp minimum length;(2) 50% or higher GC content; (3) 0.60 or higher ratio of observed/expected dinucleotides CpG.

Finally, three regions from CpG islands of HMGCR, one from that of SCAP, three from that ofSREBF1/2 and four from that of TBL2 were selected and sequenced.

The percentage of methylated cytosine relative to the total tested cytosines were calculated as the methylation level at each CpG site. The average methylation level of all detected CpG sites in the gene was taken as the average methylation level.

## Statistical analysis

Normality of parameters was assessed by Shapiro–Wilk test. The measurement data which meet the normality assumption are shown as the means±SD, and the differences between the hyperlipidemia group and control group were assessed using an independent-sample t-test. For data did not meet the normality assumption, they were described as median (interquartile range) and compared with Mann–Whitney U test. The non-parameters were compared with Fisher’s exact test.

Methylation levels of different genes were compared between patients group and control group by using Mann–Whitney U test. Mann–Whitney U test for the abundance of each methylated haplotype, screening for methylated haplotypes with significant differences in abundance. Logistic regression analyses with effect ratios (OR and 95% CI) were used to assess the contribution of the major risk factors to hyperlipidemia.

Data were analyzed using IBM SPSS Statistics Version 22.0 (Armonk, NY: IBM Corp.). A two-tailed value of P ＜ 0.05 was considered statistically significant.

## Outcome(s)

The average methylation levels of CpG sites measured within HMGCR, SCAP, SREBF1/2 were not significantly correlated to hyperlipidemia, while those within TBL2 were significantly different.

The methylation haplotype with significant abundance of TBL2 gene are tcttttttttt (p=0.034),ctttttttcct (p=0.025),ctctttctttt (p=0.040),ccttttttttt (p=0.028),tctttttttttttttt (p=0.019),tttttttttttttttc (p=0.031) and tttttttttttttctt (p=0.015).

## Contact details for further information

Li Yang.

Xinjiang Medical University Affiliated First Hospital

CHINA.

Lynju2010@126.com.

## Organizational affiliation of the study

Department of Cardiology, First Affiliated Hospital of Xinjiang Medical University, Urumqi, China.

Xinjiang Key Laboratory of Cardiovascular Disease Research, Urumqi, China.

## Anticipated completion date

20 June 2019

## Funding sources

This work was supported by NSFC grant number 2017E0269.

## Conflicts of interest

None

**Contributor ship statement**

Li Yang and Shuai Liu contributed equally to this work. Li Yang and Shuai Liu were responsible for the statistical analysis and wrote this paper. Yong-Tao Wang and Han Min provided the database. Dilare Adi and Xiao-Mei Li were responsible for collecting clinical data. Zhen yan Fu and Yi-Ning Yang revised the paper critically for important intellectual content. Yi-Tong Ma is accountable for all aspects of the work and funding.

## Data sharing statement

The data sets generated and analyzed during the current study are available from the corresponding author upon reasonable request.

## Language

English

## Country

China
