# Supplementary material for: TBL2 methylation is associated with hyper-low-density lipoprotein cholesterolemia: a case-control study
Source: Lipids Health Dis. 2020 Aug 18;19:186. doi: 10.1186/s12944-020-01359-8 (PMC7433086; doi:10.1186/s12944-020-01359-8)
Supplement: Supplementary file 2 — Additional file 2: Appendix 2. Approval report of the Medical Ethics Committee of the First Affiliated Hospital of Xinjiang Medical University [file 12944_2020_1359_MOESM2_ESM.zip › Appendix2.docx]

## Approval report of the Medical Ethics Committee of the First Affiliated Hospital of Xinjiang Medical University

Research project name：Variation and function of key genes for cholesterol absorption in patients with metabolic syndrome in different ethnic groups in Xinjiang

Researchers：Yi-Tong Ma

Voting results：Number of voters：6 Agreed number：6

Research time：2014-01-01-2018-12-31

Approval comments：

Please follow the relevant laws, regulations and regulations of our country (SFDA "Guidelines for Quality Control of Clinical Trials" (2003), "Clinical Test Regulations for Medical Devices" (2004), WMA "Helsinki Declaration" and CIOMS "International Ethics Guide for Human Biomedical Research", Ministry of Health, “Ethical Review of Biomedical Research in Persons (Trial) (2007)”) and the approved protocols and informed consent of the Ethics Committee to protect the health and rights of the subjects. If the research plan or informed consent is revised, it should be reported to the ethics committee in time.
