# Supplementary figures and images for: TBL2 methylation is associated with hyper-low-density lipoprotein cholesterolemia: a case-control study
Source: Lipids Health Dis. 2020 Aug 18;19:186. doi: 10.1186/s12944-020-01359-8 (PMC7433086; doi:10.1186/s12944-020-01359-8)

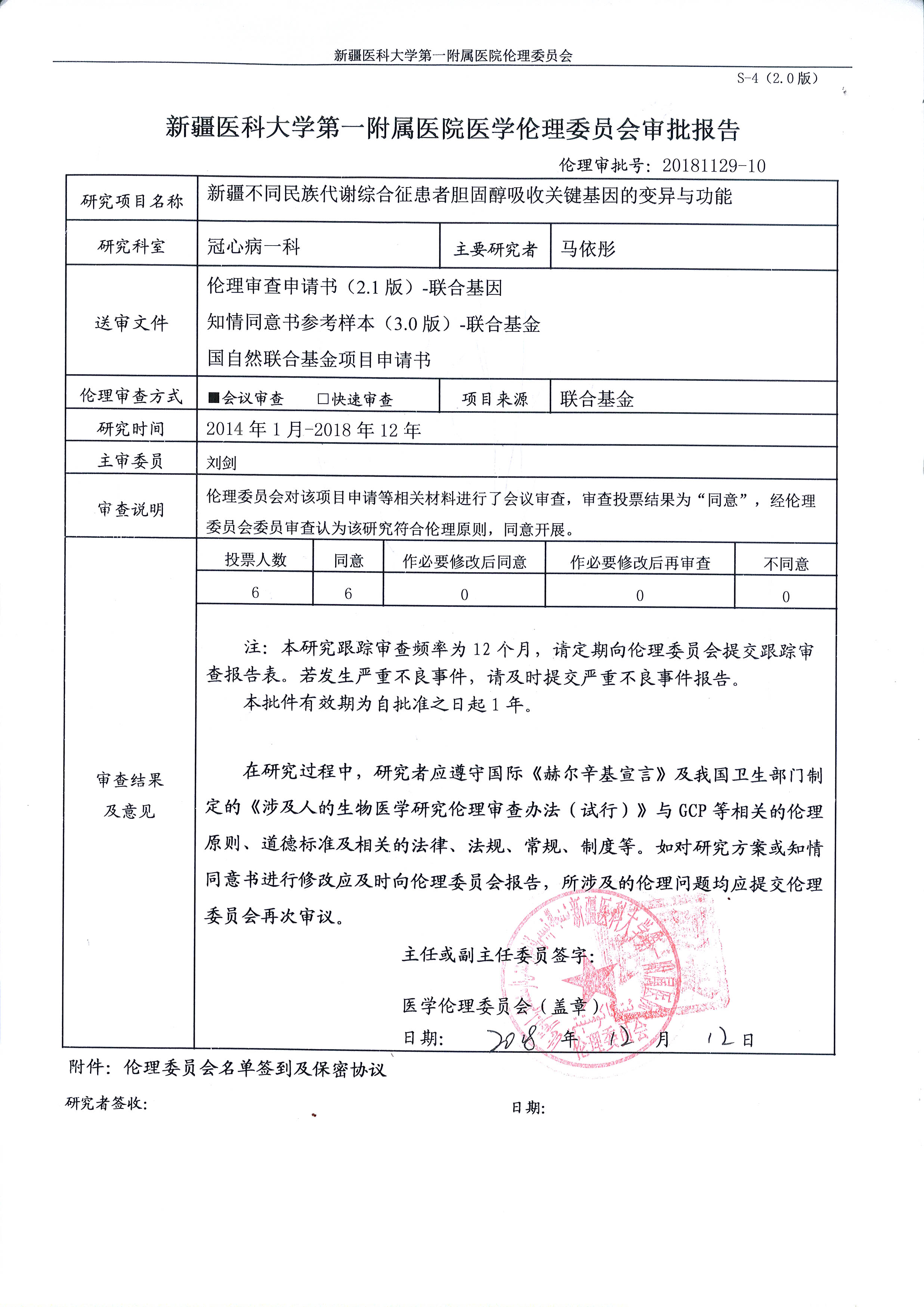

Supplement: Supplementary file 2 — Additional file 2: Appendix 2. Approval report of the Medical Ethics Committee of the First Affiliated Hospital of Xinjiang Medical University [file 12944_2020_1359_MOESM2_ESM.zip › Appendix2.tif]
